# The impact of PIT tags on the growth and survival of pythons is insignificant in randomised controlled trial

**DOI:** 10.7717/peerj.11531

**Published:** 2021-06-30

**Authors:** Patrick L. Taggart, Stephen Morris, Charles G.B. Caraguel

**Affiliations:** 1 School of Animal and Veterinary Science, The University of Adelaide, Roseworthy, SA, Australia; 2 Vertebrate Pest Research Unit, NSW Department of Primary Industries, Queanbeyan, NSW, Australia; 3 NSW Department of Primary Industries, Wollonbar, NSW, Australia

**Keywords:** Ecology, Herpetology, Python, Traceability, Passive integrated transponder, PIT, Growth, Survival, Randomised controlled trial, Snake

## Abstract

Individual identification is fundamental to the study of captive and wild animals but can have adverse impacts if the method of identification is inappropriate for the species or question of interest. We conducted a randomised controlled trial to test whether passive integrated transponder (PIT) tags reduced the growth or survival of pythons. We randomly allocated 200 captive-bred Burmese python (*Python bivittatus*) hatchlings into two groups, tagged versus untagged. Hatchlings were individually identified using a combination of PIT tags and unique colour patterns, and their mass, snout-vent length (SVL) and body condition measured at 9, 73, 134, 220, 292 and 385 days of age. We recorded the date of all mortalities. Python morphometrics and their rate of change increased or fluctuated non-linearly with age. The impact of PIT tagging on python body mass and body mass growth over the 376 day study period was insignificant. PIT tagging additionally had an insignificant impact on python survival. However, we found minor differences in SVL growth between tagged and untagged pythons. These differences peaked at approximately 0.5 mm/day and appeared to drive similar, but more pronounced, differences between tagged and untagged pythons in their rate of change in body condition; peaking at approximately 3–4 g/day. While we cannot be certain that these small differences are, or are not, biologically meaningful, they nonetheless appear to be short-term and readily resolved. Unsurprisingly, the strongest driver of python growth was their age, with growth rapidly increasing or highest amongst younger snakes for all measures of size. Python sex was associated with their body mass and survival, with higher mass but lower survival amongst females. Python size at hatching did not impact on their growth or survival. Our results confirm that PIT tags are a valuable and effective tool for the identification and tracking of captive pythons, and snakes generally, and meet high safety and animal welfare standards.

## Introduction

Animal tagging and identification is fundamental to the longitudinal study of both captive and wild animals. The individual identification of wild animals is particularly relevant to capture-mark-recapture (CMR) experiments within the field of ecology. CMR uses a time series of captures and subsequent recaptures of marked individuals to estimate population characteristics such as abundance, density, survival or recruitment (Amstrup, McDonald & Manly, 2010). However, prior to the application of any particular technique for the individual identification of animals, it is important to consider if and how the chosen technique may affect the study species (Silvy, Lopez & Peterson, 2005). Important considerations might include; (1) will the identification technique cause pain or decrease survival, (2) will the technique affect the animal’s health, reproduction, movement patterns, or behaviour, and (3) is the technique sufficiently durable to last until the completion of the study? With these considerations in mind, we tested if passive integrated transponder (PIT) tags influenced the growth or survival of Burmese pythons (*Python bivittatus*).

The Burmese python is the second most frequently traded python in South-East Asia (Kasterine et al., 2012). Approximately 150,000 Burmese python skins are legally traded annually, with evidence of some illegal trade occurring (Kasterine et al., 2012; Nossal et al., 2016). Skins are primarily purchased by the fashion industry to produce exotic leather items but are also used in the production of Chinese musical instruments. Other python parts, such as meat and gall bladders, are also sold (Kasterine et al., 2012). This industry is particularly important in developing countries, and provides opportunities for employment, income diversification, and poverty alleviation (Nossal et al., 2016).

The vast majority of exports of Burmese pythons are reported to be sourced from captive-breeding facilities (Kasterine et al., 2012). Yet, for closely related species, such as the reticulated python (*Python reticulatus*) large numbers of individuals are harvested from the wild. While the large annual harvest of these wild, large-bodied reptiles is thought to be sustainable, captive-bred animals are often favoured by the high-end leather industry based on the perception that captive-bred pythons have higher sustainability and animal welfare credentials (Natusch & Lyons, 2014; Natusch et al., 2019; Natusch et al., 2016). However, concerns have been raised that snakes marketed as captive-bred may, in fact, be wild-caught, and laundered through captive-breeding facilities under the guise of being captive-bred (Kasterine et al., 2012). Although subsequent work has shown that many pythons are genuinely bred in captivity, this concern remains (Natusch & Lyons, 2014). There is currently no robust assurance system in place to facilitate differentiating between captive-bred and wild-caught pythons, which does little to temper these concerns.

Several methods for differentiating captive-bred and wild-caught reptiles have been suggested, including assessment of their general health, appearance and behaviour, branding, evidence of eggshells from hatchlings, the breeding of non-natural colour morphs, presence of parasites, genetics, isotopic or elemental markers, and PIT tagging (Lyons & Natusch, 2011; Lyons & Natusch, 2015; Natusch et al., 2017). Each of these methods have inherent advantages, although none are perfect. An ideal method for differentiating between captive-bred and wild-caught pythons should meet the following criteria described in Lyons & Natusch (2015), including; (1) timely to implement, (2) cost efficient, (3) suitable for live animals, parts, and derivatives, (4) scalability, (5) labour efficient, and (6) reliable. An additional major benefit provided by some of the listed methods for differentiating captive-bred from wild-caught reptiles, but not considered by Lyons & Natusch (2015), is the ability of some methods to identify individual animals and allow them to be tracked throughout their production lifecycle. This can facilitate production management, trend recognition, increased transparency, and the development of optimal farm management strategies, thereby promoting industry advancement and improvement in animal welfare. For example, within the agricultural industry, animal identification and traceability systems are beneficial for collecting production data to support management decisions, breeding, health management, food safety, and ensuring consumer trust in food safety and quality (Augsburg, 1990; Elbakidze, 2007; Van Rijswijk & Frewer, 2008; Wang et al., 2010).

The best animal identification and traceability systems allow the user to track individual animals through time from birth to death. Branding, genetic identification and PIT tagging are the only methods of differentiating captive-bred from wild-caught reptiles suggested by Lyons & Natusch (2015) that would also allow individual animal tracking and provide additional animal management benefits to python farmers. Branding snakes is relatively time, cost, and labour efficient and has the potential for scalability, but is only suitable for use in live animals and may occasionally be unreliable if brands fade over time (Shine et al., 1988; Winne et al., 2006). Where animal numbers are large, branding may additionally be limited by the number of unique combinations of brands that can be applied. However, this method of marking animals would likely be considered inappropriate by fashion labels wishing to maintain high ethical standards due to it involving physically marking the skin of the animal such that a scar is formed. Unlike branding, genetic analysis can identify an unlimited number of individuals, is highly reliable, ethical, and suitable for use in live animals, as well as body parts and derivatives. But, genetic analysis is expensive, labour and time intensive to implement, and has limited scalability. PIT tagging is relatively time, labour, and cost efficient, it has the potential for scalability, can identify an unlimited number of animals, and is generally considered to be highly ethical (Mellor, Beausoleil & Stafford, 2004). Yet, PIT tagging is primarily used in live animals and can occasionally be unreliable if tags are expelled (Roark & Dorcas, 2000). Furthermore, the degree to which PIT tagging impacts the health, behaviour, or welfare of pythons is unknown, despite evidence suggesting the impacts of PIT tagging in other taxa are minimal (Ombredane, Bagliniere & Marchand, 1998; Ott & Scott, 1999). It is therefore important to determine if there are any significant negative impacts of PIT tagging on the growth or survival of pythons before this identification technique is adopted, or to ensure it is appropriate, for the study or tracking of pythons in captive facilities or ecological studies (Leuenberger et al., 2019; Lind, McCoy & Farrell, 2018; McKenzie et al., 2021).

To address this knowledge gap, we conducted a randomised controlled trial to test if PIT tags influenced the growth or survival of captive-bred Burmese pythons. We predicted that the growth and survival of tagged pythons would be indistinguishable from untagged animals, based on results in other species (Ombredane, Bagliniere & Marchand, 1998; Ott & Scott, 1999). We accordingly predicted that PIT tagging would be an appropriate method of individually identifying pythons, whilst simultaneously providing additional benefits associated with the individual identification of snakes within a production context.

## Materials & Methods

This report follows the CONSORT checklist guidelines for the transparent reporting of trials and received animal ethics approval through The University of Adelaide (permit number: S-2018-084) (Schulz, Altman & Moher, 2010).

### Trial design and morphological measurements

We conducted a parallel group randomised controlled trial to investigate the impact of PIT tags on the growth and survival of Burmese pythons. Two hundred python hatchlings were sourced from, housed and monitored within a commercial python farm in Cù Chi, Vietnam over a period of approximately 13 months. Mr. Jose Danvila, director of Verdeveleno, granted permission to work on private python farms in Vietnam. Hatchlings from eight different clutches were allocated into two groups (tagged vs untagged animals) of 100 individuals each following a systematic random allocation protocol. This sample size of 100 python hatchlings in each group was selected arbitrarily and no blinding to group allocation occurred.

**Figure 1:**
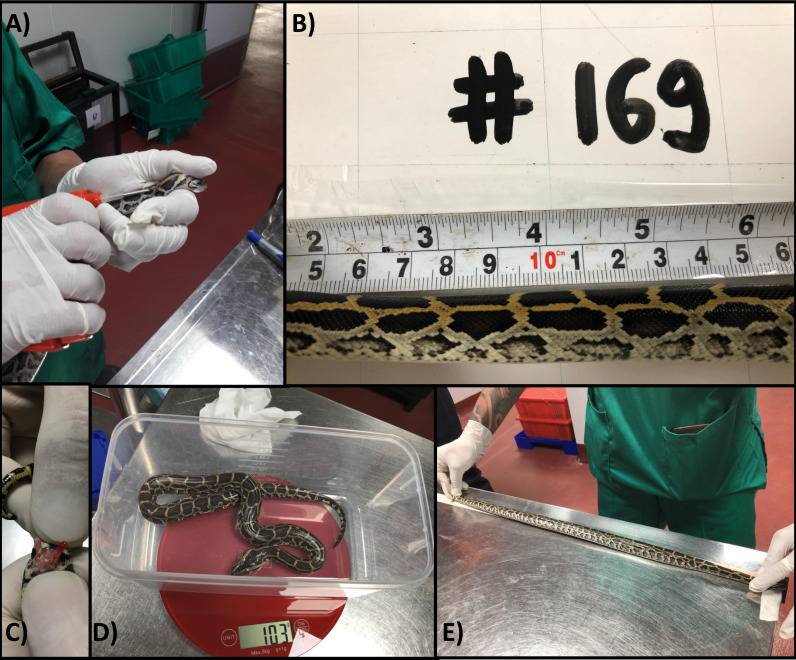
Individually identifying and measuring python hatchlings. (A) Insertion of passive integrated transponder tag into dorsal cervical region of python hatchling; (B) photographing the unique colour patterns of the right side cervical region of a python hatchling and allocation photographic identification to a unique animal number; (C) sexing of python hatchlings by everting hemipenes, male pictured; (D) measurement of python hatchling body mass (g); (E) measurement of python hatchling snout-vent length (mm).

Hatchlings in the tagged group were identified by inserting a unique PIT tag (13 × 2 mm) subcutaneously into the dorsal cervical region by one of three different farm workers (Fig. 1A). Farm workers were trained in PIT tag insertion using a hand-held insertion device prior to conducting the procedure. Sterile PIT tag insertion needles were replaced after each use, but python skin was not disinfected prior to tag insertion and insertion points were not sealed. All trial pythons were additionally identified by photographing the unique colour patterns of their left side cervical region (Bauwens, Claus & Mergeay, 2018) (Fig. 1B). This allowed us to identify untagged individuals throughout the trial and tagged individuals in the case that their PIT tag was expelled or malfunctioned. Python hatchlings were housed in groups of 25 in eight separate wire mesh pens (e.g., 12/13 tagged/untagged pythons per pen) where they remained for the duration of our study. Tagging and pen allocation occurred on a single day. However, to source enough hatchlings to reach the target sample size we used pythons that had hatched on either the same day or up to two days earlier.

We recorded sex (by everting hemipenes), body mass (to the nearest g) and snout-to-vent length (SVL; to the nearest mm) for each hatchling at 9, 73, 134, 220, 292 and 385 days of age (Figs. 1C–1E).

All python hatchlings were raised in the same manner, following the standard farm procedures for feeding, watering, cleaning, and handling. All pythons are fed individually for their duration of life on the farm. This gives farmers greater control over the amount of food that each python receives, prevents conflict between pythons over food, and better ensures food is evenly distributed among all pythons. Pythons less than 200 g were fed whole juvenile quail or small rats approximately every four days. Pythons ranging from 200–2,000 g were fed approximately 20–30% of their body mass in grams of whole dead chicken hatchlings every 5–6 days. Pythons ranging from 2,000–6,000 g were fed approximately 50% of their body mass in grams of dead chicken (whole or head/neck) every 5–6 days. While pythons greater than 6,000 g were fed approximately 30% of their body mass in grams of dead chicken every 7–8 days. We recorded the date of mortality for all pythons found dead prior to the end of the trial.

### Data analysis

All statistical analyses were performed in R version 4.0.2 (R Core Team, 2020). Plots were created in the *ggplot2* package (Wickham, 2016). We included data from all 200 pythons in all analyses. All data and R code is accessible from PeerJ in consultation with the authors.

#### Body condition index

We calculated the scaled mass index as a measure of each python’s body condition (Peig & Green, 2009). Body condition was calculated across all pythons and all time points to facilitate the comparison of body condition through time. In our application, the scaled mass index computes the body mass each python would have at a fixed SVL. Relative to other body condition indices, the scaled mass index more reliably reflects changes in an animal’s mass and length as body size changes and growth occurs (Peig & Green, 2010).

#### Confirmation of successful random allocation

We compared the characteristics of hatchlings between the tagged and untagged group to assess the success of our random allocation procedure. We compared the relative number of tagged and untagged hatchlings between sexes, clutches and pens using a Chi-squared test. In a similar manner, we compared body mass, SVL, and body condition between tagged and untagged hatchlings using a two-sample *t*-test. We additionally compared the total number of pythons tagged by each farm worker.

#### Python growth

We constructed three sets of generalised additive mixed models (GAMM) to investigate the association between body mass, SVL, and body condition, and a set of predictor variables. All models were constructed with a Gaussian family specification within the *mgcv* package, with smoothing parameters estimated using restricted maximum likelihood (Wood, 2012). We included PIT tag status (tagged/untagged) and sex as a discrete fixed effect in all models as our experiment was specifically designed to test the effect of tagging on python growth. We additionally included a discrete fixed effect for python sex. We included a penalised thin plate regression spline for python age to capture non-linear changes in growth (Wood, 2003). We allowed the spline component of the models to vary according to PIT tag status and sex. To account for repeated measurements on individual pythons through time we included a random intercept and slope for individuals.

For all python growth models, we assessed model parsimony using AIC. We considered a model to have substantial support if ΔAIC ≤ 2, and where multiple models had equivalent ΔAIC values we chose the model with the fewest parameters (Burnham & Anderson, 2002; Pedersen et al., 2019; Wood, Pya & Säfken, 2016). We used model diagnostics produced by *gam.check()* in *mgcv* to assess model assumptions and fit (Wood, 2017).

From our most parsimonious model of body mass, SVL, and body condition, we predicted the growth curves on a daily basis and calculated the first derivative using finite differences in the *gratia* package (Simpson, 2018). This allowed us to describe python growth rates, in addition to the association between each of our three measures of python size (body mass, SVL and body condition) and PIT tag status, sex and time/python age.

To test if the size of python hatchlings influenced their size at subsequent time points, we calculated Pearson correlation coefficients for python size at hatching and their size at each subsequent time point. If python size at hatching influenced their subsequent size, we expected to see a stable and consistent association between their size at hatching and subsequent size measurements. However, if the size of python hatchlings did not influence their subsequent size, we expected that the association between hatchling size and subsequent size would erode or be inconsistent through time.

#### Python survival

We used generalised linear mixed models to investigate the association between survival and a series of predictors. Python survival was defined across our whole study period; 0 = individual did not survive whole study period, 1 = individual survived whole study period. Survival models were constructed with a binomial family specification within *glmmTMB* (Brooks et al., 2017) and following the data exploration guidelines provided by Zuur, Ieno & Elphick (2010).

When modelling survival, we included random intercepts for pen and clutch. We included PIT tag status as a fixed effect in all survival models as our experiment was specifically designed to test the effect of tagging on python survival. We additionally included sex and hatchling size (body mass, SVL, and body condition) as fixed effects to test their effect on survival. For descriptive purposes, we calculated Kaplan–Meier survival time curves within the *survival* package (Kaplan & Meier, 1958; Therneau, 2015).

We assessed parsimony for survival models using AICc as described above. We checked model residuals within the *DHARMa* package (Hartig, 2020).

## Results

Python hatchlings comprised 105 females and 95 males. Forty six males and 54 females were tagged and 49 males and 51 females remained untagged. Two pythons lost their PIT tag shortly after they were implanted, within three days, which was presumed to have exited the body through the hole created during the procedure. Both pythons were re-implanted with the same PIT tag and no other tags were lost or expelled by pythons for the remainder of our study. This equated to a PIT tagging success rate of 98% for the initial insertion of tags and a retention rate of 100% for the remainder of the study.

### Confirmation of successful random allocation

We found no difference in the distribution of sexes (*χ*^2^ (DF = 1, *N* = 200) = 0.18, *p* = 0.77), clutches (*χ*^2^ (DF = 7, *N* = 200) = 0.17, *p* = 1.00) or pens (*χ*^2^ (DF = 7, *N* = 200) = 0.32, *p* = 1.00) between tagged and untagged pythons at the beginning of our study. Similarly, we found no difference in hatchling body mass (*t* = 1.19, *df* = 198.0, *p* = 0.24), SVL (*t* = 0.30, *df* = 197.5, *p* = 0.76) or body condition (*t* = 0.64, *df* = 197.8, *p* = 0.52) between tagged/untagged groups at the beginning of our study. All farm workers tagged and handled a similar number of pythons (worker #1 tagged 35, worker #2 tagged 33, worker #3 tagged 32).

### Python growth

**Figure 2:**
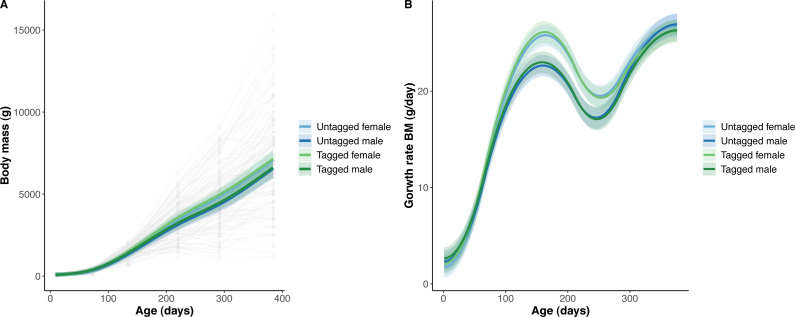
Python body mass (BM) and body mass growth across age (days). (A) Predicted python BM (solid lines) with 95% confidence intervals (faded colour ribbons) across age by PIT tag status and sex, and observed individual growth curves (faded grey points and lines). (B) Predicted python BM growth rate (solid lines) with 95% confidence intervals (faded colour ribbons) across age by PIT tag status and sex.

**Figure 3:**
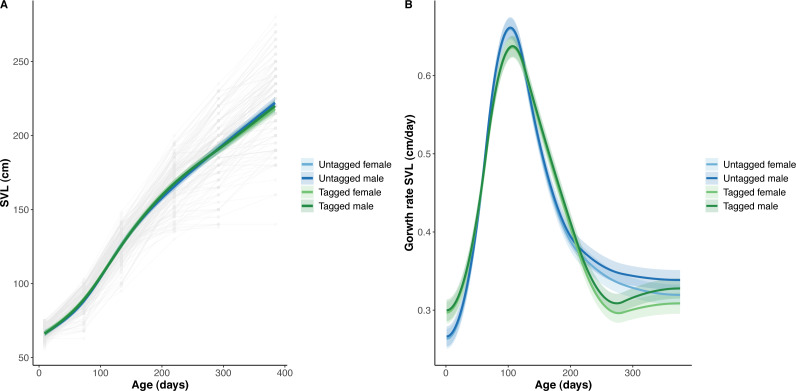
Python snout to vent length (SVL) and snout to vent growth across age (days). (A) Predicted python SVL (solid lines) with 95% confidence intervals (faded colour ribbons) across age by PIT tag status and sex, and observed individual growth curves (faded grey points and lines). (B) Predicted python SVL growth rates (solid lines) with 95% confidence intervals (faded colour ribbons) across age by PIT tag status and sex.

**Figure 4:**
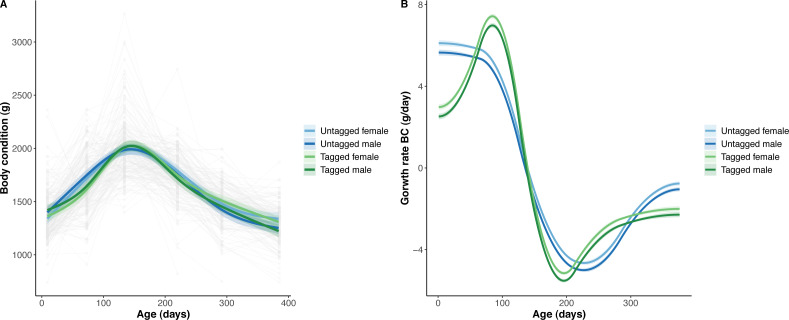
Python body condition (BC) and the rate of change in body condition across age (days). (A) Predicted python BC (solid lines) with 95% confidence intervals (faded colour ribbons) across age by PIT tag status and sex, and observed individual growth curves (faded grey points and lines). (B) Predicted rate of change in python BC (solid lines) with 95% confidence intervals (faded colour ribbons) across age by PIT tag status and sex.

The overall effect of PIT tag status on all measures of python size, and body mass growth, was insignificant (Figs. 2–4). However, we found small (≤ 0.5 mm/day) and alternating differences in SVL growth between tagged and untagged pythons. For example, SVL growth was greater for tagged relative to untagged snakes between approximately 0 and 60 days of age, lower between 60 and 130 days of age, greater between 130 and 200 days of age, and then finally lower between 200 and 300 days of age. These alternating differences in SVL growth between tagged and untagged pythons appeared to drive similar, but more pronounced, alternating differences between tagged and untagged pythons in the rate of change in body condition. Differences in the rate of change in body condition between tagged and untagged pythons were initially approximately 3 g per day, but declined to 1−1.5 g per day by 50 days of age and thereafter remained low for the remainder of our trial.

**Figure 5:**
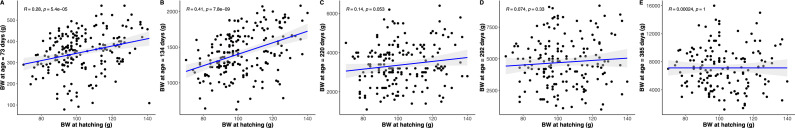
Pearson correlations between hatchling body mass (BM) and subsequent python body mass at each measurement time point. Similar figures were produced, but are not presented, showing an equivalent inconsistent or eroding relationship between hatchling snout to vent length and body condition, and subsequent python snout to vent length and body condition, respectively, as age increases.

The strongest driver of python size and growth was their age, irrespective of the measure of size used (Figs. 2–4). Python body mass and SVL, and the variability in these measures, consistently increased through time, but most rapidly in young snakes (Figs. 2–3). In contrast, python body condition showed a hill-shaped pattern, increasing rapidly in young snakes until approximately 130 days of age, before decreasing for the remainder of our study (Fig. 4). These patterns were reflected in growth curves. Growth in body mass and SVL increased rapidly until approximately 100–130 days of age, after which growth in body mass began to plateau and growth in SVL began to decrease. In contrast, the rate of change in body condition started high, decreased between approximately 70–100 days of age and 200 days of age and then plateaued thereafter. While python growth was greatest when they were young and small, the size of pythons at hatching did not appear to influence their size at subsequent time points/as adults (Fig. 5). This was demonstrated by an inconsistent or eroding relationship between hatchling size and subsequent python size as age increased for all measures of size (body mass, SVL, and body condition).

Python growth was also associated with their sex. This was evident in their body mass which showed females diverging, and increasing, in size relative to males from approximately 100 days of age onwards (Fig. 2).

Our models for both python body mass and SVL explained a particularly large amount of the deviance in our data, >95% (Tables S1–S2). For both python body mass and SVL the inclusion of random slopes for individual pythons through time accounted for a large amount of the variability in these measures of size (i.e., individual python growth effect). However, the inclusion of random intercepts for individual pythons accounted for minimal variability in these measures of size due to the relatively minimal variation in body mass and SVL at hatching and large variation in these measures at later time points throughout our study. In contrast, our model for python body condition explained much less, but still a reasonable amount, of the deviance in our data, approximately 60% (Table S3). The inclusion of random intercepts for individual pythons accounted for a reasonable amount of the variability in body condition and the inclusion of random slopes for individual pythons through time was not supported.

To enable us to plot and demonstrate the estimated effects of tagging and python sex through time, we retained separate penalised thin plate regression splines for tagged and untagged, and male and female, pythons through time/across ages in our final models for all measures of size; although they were not always supported based on AIC.

#### Python survival

**Figure 6:**
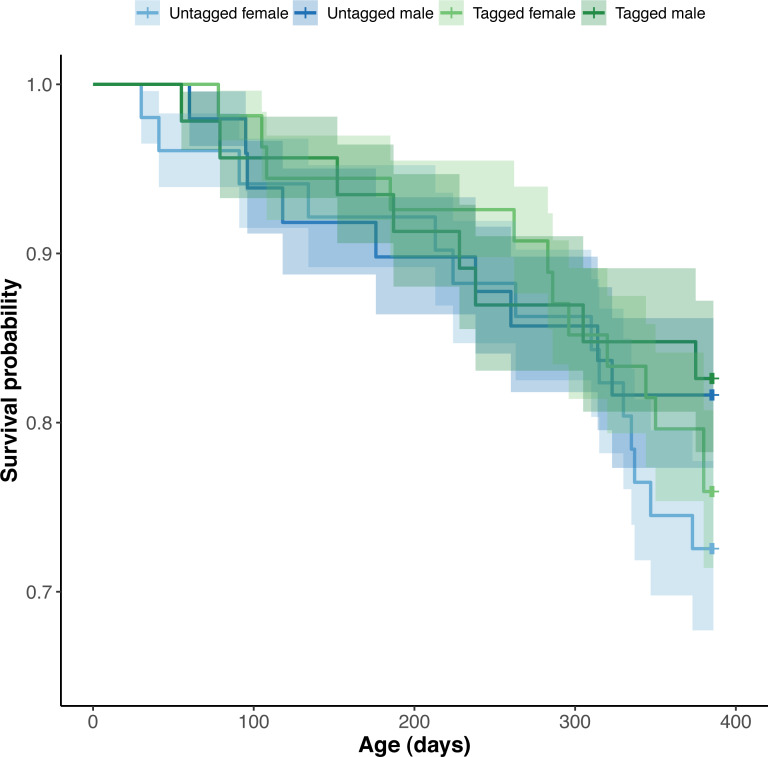
Estimated Kaplan–Meier survival curves and their respective 95% confidence intervals for tagged and untagged, and male and female pythons across age (days).

Python mortality occurred between all successive snake measurement points (Fig. 6). Four pythons died between 9 and 73 day of age, nine between 74 and 134 days of age, five between 135 and 220 days of age, nine between 221 and 292 days of age and 17 between 292 and 385 days of age. We were unable to obtain detailed information on the cause of python death.

The effect of PIT tag status on python survival was insignificant (OR: 1.18; 95% CI: 0.88, 1.57) (Fig. 6). Similarly, we found an insignificant effect of python size at hatching on their survival (association between body condition at hatching and survival—OR: 1.00, 95% CI [1.00, 1.00]; association between SVL at hatching and survival—OR: 1.01, 95% CI [0.95, 1.06]). Python survival appeared to be driven by their sex. Female pythons had decreased survival relative to males (OR (males:females): 1.80; 95% CI [1.33, 2.44]), resulting in an equal number of male and female pythons at the end of our study, despite there initially being ten more female pythons than males at the start of our study (Fig. 6). We found no evidence for an effect of hatching body mass on python survival. Variance in survival attributable to python clutch (random intercept variance: 0.17; random intercept standard deviation: 0.41) and pen (random intercept variance: 0.53; random intercept standard deviation: 0.73) was minimal.

Our model selection process and information relating to the goodness of fit for all growth and survival models is summarised in TablesS1–S4.

## Discussion

Passive integrated transponder (PIT) tags have been used to mark reptiles for many years and can be an effective means of individually identifying many species (Ferner, 2007). However, their use in snakes, and particularly in pythons, compared to other groups of reptiles is less well-known. As a consequence, some authors have suggested that before being used researchers should carry out preliminary work, especially on snake species, to determine whether PIT tags are retained, and whether they will affect the individual’s growth or survival (Plummer & Ferner, 2012). Our results confirm that PIT tags are a valuable and effective tool for the identification and tracking of captive pythons, and snakes generally, and meet high safety and animal welfare standards.

PIT tagging had an insignificant impact on all measures of python size (body mass, SVL, and body condition) and their survival, and only small impacts on their growth rates. These results are consistent with previous studies in which PIT tagging had no influence on the growth or survival of other species of reptile (Jemison et al., 1995; Keck, 1994; Paquet et al., 2011). However, we found minor differences in SVL growth and the rate of change in body condition between tagged and untagged pythons. For the majority of our study, differences in SVL growth and the rate of change in body condition were small and only reached peaks of 0.5 mm and 3–4 g per day, respectively. If such differences continued throughout the life of the pythons, they may be biologically important, although over the duration of our study we suggest these differences were of minor biological consequence. At worst, these differences may represent a short-lived reduction in the rate of change in body condition immediately post tagging due to stress or other health impacts induced by the tagging procedure. Although we were unable to identify why this effect may occur, it is nonetheless short-lived.

PIT tagging and retention rates in our study approached 100%. These results are an improvement on some previous studies where a high proportion of tags implanted subcutaneously into snakes were suggested to have been expelled from the body (Roark & Dorcas, 2000). There are multiple possible explanations why PIT tag retention rates in our study were higher than have previously been reported. The method and location of PIT tag insertion in our study may have been optimal for minimising tag migration within the body. However, this seems unlikely given that Roark & Dorcas (2000) also inserted PIT tags into the neck region of corn snakes (*Pantherophis guttatus*) and found that 66% of snakes moved their tag to the mid-body region or beyond and 53% of snakes expelled their tag at least once. PIT tag migration may also be reduced in captive snakes due to their reduced movement compared to wild snakes. This may decrease the probability that tags exit through the insertion point or are expelled otherwise by the snake. While the results of Roark & Dorcas (2000) were also in captive snakes, it is difficult to determine if or how the movement of the snakes in their study may have been limited by their housing and maintenance. Alternatively, PIT tag movement and expulsion may be more profound in some snake species, with smaller species, or species for which the tag is a more significant burden, expending greater energy to move or expel it from their body.

We envisage three major challenges with the widespread use of PIT tags within a captive-breeding setting; (1) the inability of PIT tags to identify or trace python parts or derivatives, (2) the inability of PIT tags to completely prevent the laundering of wild animals through captive facilities, and (3) the long-term capture, storage and analysis of PIT tag information. Whilst the technology exists to capture and store large quantities of digital PIT tag information, we appreciate that python farming and breeding often occurs in developing regions where resources may be limited or the quantitative skills necessary to handle and analyse such data may be lacking.

Python age was the strongest predictor of their size and growth in our study. Young pythons in our study showed rapidly increasing body mass and SVL growth, before growth in body mass plateaued and growth in SVL decreased at approximately 100–130 days of age. This plateau and decrease in body mass and SVL growth, respectively, corresponded with the start of the cooler dry season when python feed intake and growth is known to reduce. Similarly, the rapidly increasing growth in young animals may be explained by their food intake, which is more frequent relative to older animals—although we did not explicitly measure their food consumption to determine if they receive a greater volume of food relative to their size. Rapidly increasing growth amongst smaller, and potentially younger, snakes has also been recorded in the wild (Brown, Madsen & Shine, 2017; Madsen & Shine, 2001; Webb, Brook & Shine, 2003). In wild snakes this may help to reduce an individual’s predation risk, their vulnerability to starvation or stress, increase their capacity to swallow larger prey items, or to obtain a more stable thermal mass (Seebacher & Shine, 2004; Webb, Brook & Shine, 2003).

Python growth in our study was also associated with their sex. While we cannot discount the possibility of sex differences in growth being driven by sex differences in food intake, we suggest this is unlikely and note that our results are consistent with the known sexual size dimorphism in this species, and closely related species (Natusch & Lyons, 2014; Natusch et al., 2019). Our results contradict the notion that pythons do not diverge in size until post sexual maturity (Natusch & Lyons, 2014). The development of sexual size dimorphism in snakes is suggested to generally follow one of two trajectories; (1) it can exist amongst neonates and persist, or be magnified, into adulthood (King et al., 1999); or (2) it may not exist in neonates but develop post sexual maturity (Beaupre, Duvall & O’Leile, 1998; Taylor & Denardo, 2005). Our results show that male and female Burmese pythons begin to diverge in body mass at approximately 100 days of age, when they are only 1000 g or less, with females returning to a growth rate equivalent to that of males by approximately 330 days of age and approximately 5000 g body mass. This suggests that sexual size dimorphism in this species, while non-existent as hatchlings, begins to develop prior to sexual maturity, or at least develops with the development of sexual maturity, and is evident by the time snakes are sexually mature. We would expect that close relatives of Burmese pythons also show a similar pattern in the development of sexual size dimorphism.

Python sex also appeared to influence their survival, with lower survival amongst females. Reduced female survival in snakes has previously been suggested to be the norm, due to costs associated with reproduction, although reduced survival amongst males has been reported (Madsen & Shine, 1993; Sperry & Weatherhead, 2009). In our study, reduced female survival is unlikely to be due to higher costs of reproduction—as no individuals had reproduced. Amongst wild snakes, size can also influence survival when mortality is driven by predation or food availability and size (Forsman, 1993; Shine et al., 2001). We are uncertain why we may have observed differences in python survival between sexes as females were generally of equivalent or greater size than males. When our results are combined with those reported in the literature, snake survival appears to vary inconsistently with their size and sex, suggesting that the direction and magnitude of these effects are context dependent.

Overall, our parallel group randomised controlled trial investigated the impact of PIT tags on the growth and survival of pythons. We demonstrate that PIT tags are a safe and effective method of individually identifying pythons. Our results suggest that PIT tags hold significant potential for the management of pythons within production facilities and are a valuable method of marking wild snakes in ecological studies.

##  Supplemental Information

10.7717/peerj.11531/supp-1 Supplemental Information 1 Tables S1–S4 Model selection process and information relating to goodness of fit for growth and survival models.

10.7717/peerj.11531/supp-2 Supplemental Information 2 Raw data of python measurements

10.7717/peerj.11531/supp-3 Supplemental Information 3 R Script used for analysis

10.7717/peerj.11531/supp-4 Supplemental Information 4 ARRIVE 2.0 Checklist

## Additional Information and Declarations

### Field Study Permissions

The following information was supplied relating to field study approvals (i.e., approving body and any reference numbers): Mr Jose Danvila, director of Verdeveleno, granted permission to work on private python farms in Vietnam.

### Data Availability

The following information was supplied regarding data availability: The raw data measurements and the R script used to produce analysis/results are available in the Supplemental Files.
