# Supplementary material for: The impact of PIT tags on the growth and survival of pythons is insignificant in randomised controlled trial
Source: PeerJ. 2021 Jun 30;9:e11531. doi: 10.7717/peerj.11531 (PMC8254472; doi:10.7717/peerj.11531)
Supplement: Supplemental Information 1 — Model selection process and information relating to goodness of fit for growth and survival models. [file peerj-09-11531-s001.docx]

Table S1: Body mass – generalised additive mixed model selection process

| Model | **Model parameters** | | | | | | | | | AIC | Δ AIC | Deviance explained (%) |
| --- | --- | --- | --- | --- | --- | --- | --- | --- | --- | --- | --- | --- |
|  | Tagged | s(Age, k = 4) | s(Age, k = 5) | s(Age, k = 6) | s(Age, by = Tagged, k = 5) | Sex | s(Age, by = Sex, k = 5) | s(Individual, bs = "re") | s(Age, Individual, bs = "re") |  |  |  |
| 0 |  |  |  |  |  |  |  |  |  | 20468.74 | 2851.8 | 0.0 |
| 1 | ✓ |  |  |  |  |  |  |  |  | 20470.6 | 2853.6 | 0.0 |
| 2 | ✓ | ✓ |  |  |  |  |  |  |  | 18981.4 | 1364.5 | 74.6 |
| 3 | ✓ |  | ✓ |  |  |  |  |  |  | 18971.7 | 1354.8 | 74.8 |
| 4 | ✓ |  |  | ✓ |  |  |  |  |  | 18972.9 | 1356.0 | 74.8 |
| 5 | ✓ |  |  |  | ✓ |  |  |  |  | 18978.3 | 1361.4 | 74.8 |
| 6 | ✓ |  |  |  | ✓ | ✓ |  |  |  | 18971.7 | 1354.7 | 75.0 |
| 7 | ✓ |  |  |  | ✓ | ✓ | ✓ |  |  | 18963.6 | 1346.7 | 75.3 |
| 8 | ✓ |  |  |  | ✓ | ✓ | ✓ | ✓ |  | 18776.3 | 1159.4 | 83.3 |
| 9 | ✓ |  |  |  | ✓ | ✓ | ✓ | ✓ | ✓ | 17616.9 | 0.0 | 94.8 |

Table S2: Snout to vent length – generalised additive mixed model selection process

| Model | **Model parameters** | | | | | | | | | AIC | Δ AIC | Deviance explained (%) |
| --- | --- | --- | --- | --- | --- | --- | --- | --- | --- | --- | --- | --- |
|  | Tagged | s(Age, k = 4) | s(Age, k = 5) | s(Age, k = 6) | s(Age, by = Tagged, k = 6) | Sex | s(Age, by = Sex, k = 6) | s(Individual, bs = "re") | s(Age, Individual, bs = "re") |  |  |  |
| 0 |  |  |  |  |  |  |  |  |  | 11947.5 | 3954.6 | 0.0 |
| 1 | ✓ |  |  |  |  |  |  |  |  | 11949.2 | 3956.3 | 0.0 |
| 2 | ✓ | ✓ |  |  |  |  |  |  |  | 9131.5 | 1138.6 | 92.5 |
| 3 | ✓ |  | ✓ |  |  |  |  |  |  | 9101.8 | 1108.9 | 92.7 |
| 4 | ✓ |  |  | ✓ |  |  |  |  |  | 9093.8 | 1100.9 | 92.7 |
| 5 | ✓ |  |  |  | ✓ |  |  |  |  | 9101.8 | 1109.0 | 92.8 |
| 6 | ✓ |  |  |  | ✓ | ✓ |  |  |  | 9100.2 | 1107.3 | 92.8 |
| 7 | ✓ |  |  |  | ✓ | ✓ | ✓ |  |  | 9101.5 | 1108.6 | 92.8 |
| 8 | ✓ |  |  |  | ✓ | ✓ | ✓ | ✓ |  | 8814.0 | 821.1 | 95.7 |
| 9 | ✓ |  |  |  | ✓ | ✓ | ✓ | ✓ | ✓ | 7992.9 | 0.1 | 98.1 |
| 10 |  |  |  |  | ✓ |  | ✓ | ✓ | ✓ | 7992.9 | 0.0 | 98.1 |

Table S3: Body condition – generalised additive mixed model selection process

| Model | **Model parameters** | | | | | | | | | AIC | Δ AIC | Deviance explained (%) |
| --- | --- | --- | --- | --- | --- | --- | --- | --- | --- | --- | --- | --- |
|  | Tagged | s(Age, k = 4) | s(Age, k = 5) | s(Age, k = 6) | s(Age, by = Tagged, k = 6) | Sex | s(Age, by = Sex, k = 6) | s(Individual, bs = "re") | s(Age, Individual, bs = "re") |  |  |  |
| 0 |  |  |  |  |  |  |  |  |  | 15824.13 | 821.2 | 0.0 |
| 1 | ✓ |  |  |  |  |  |  |  |  | 15825.7 | 822.7 | 0.0 |
| 2 | ✓ | ✓ |  |  |  |  |  |  |  | 15090.1 | 87.1 | 49.3 |
| 3 | ✓ |  | ✓ |  |  |  |  |  |  | 15073.3 | 70.4 | 50.2 |
| 4 | ✓ |  |  | ✓ |  |  |  |  |  | 15047.4 | 44.5 | 51.4 |
| 5 | ✓ |  |  |  | ✓ |  |  |  |  | 15044.7 | 41.7 | 52.0 |
| 6 | ✓ |  |  |  | ✓ | ✓ |  |  |  | 15046.1 | 43.1 | 52.0 |
| 7 | ✓ |  |  |  | ✓ | ✓ | ✓ |  |  | 15035.1 | 32.1 | 52.6 |
| 8 | ✓ |  |  |  | ✓ | ✓ | ✓ | ✓ |  | 15003.0 | 0.0 | 59.1 |
| 9 | ✓ |  |  |  | ✓ | ✓ | ✓ | ✓ | ✓ | 15003.0 | 0.0 | 59.1 |

Table S4: Survival – generalised linear mixed model selection process

| Model | **Model parameters** | | | | | | | LL | K | AICc | Δ AICc | AICc Wt | Cum Wt |
| --- | --- | --- | --- | --- | --- | --- | --- | --- | --- | --- | --- | --- | --- |
|  | Tagged | Sex | Hatch BM | Hatch SVL | Hatch BC | (1 \| Clutch) | (1 \| Pen) |  |  |  |  |  |  |
| 0 |  |  |  |  |  |  |  | -632.3 | 1.0 | 1266.6 | 75.7 | 0.0 | 1.0 |
| 1 | ✓ |  |  |  |  |  |  | -631.9 | 2.0 | 1267.9 | 77.0 | 0.0 | 1.0 |
| 2 | ✓ | ✓ |  |  |  |  |  | -626.5 | 3.0 | 1259.0 | 68.0 | 0.0 | 1.0 |
| 3 | ✓ | ✓ | ✓ |  |  |  |  | -626.4 | 4.0 | 1260.8 | 69.9 | 0.0 | 1.0 |
| 4 | ✓ | ✓ |  | ✓ |  |  |  | -623.9 | 4.0 | 1255.9 | 64.9 | 0.0 | 1.0 |
| 5 | ✓ | ✓ |  | ✓ | ✓ |  |  | -620.0 | 5.0 | 1250.0 | 59.0 | 0.0 | 1.0 |
| 6 | ✓ | ✓ |  | ✓ | ✓ | ✓ |  | -597.7 | 6.0 | 1207.4 | 16.5 | 0.0 | 1.0 |
| 7 | ✓ | ✓ |  | ✓ | ✓ | ✓ | ✓ | -588.4 | 7.0 | 1190.9 | 0.0 | 1.0 | 1.0 |
| LL = log likelihood; K = number mode parameters; Wt = weight; Cum = cumulative | | | | | | | | | | | | | |
